# *GoFish* : a foray into open‐source, aquatic behavioral automation

**DOI:** 10.1111/jfb.15937

**Published:** 2024-09-23

**Authors:** Victor Ajuwon, Bruno Cruz, Tiago Monteiro

**Affiliations:** ^1^ Department of Psychology University of Cambridge Cambridge UK; ^2^ NeuroGears Ltd. London UK; ^3^ Domestication Lab, Konrad Lorenz Institute of Ethology, Department of Interdisciplinary Life Sciences University of Veterinary Medicine Vienna Vienna Austria; ^4^ William James Center for Research University of Aveiro Aveiro Portugal; ^5^ Department of Education and Psychology University of Aveiro Aveiro Portugal; ^6^ Present address: Allen Institute for Neural Dynamics Seattle Washington USA

**Keywords:** automatic feeder, behavioral automation, bonsai, fish behavior, fish cognition, *GoFish*, operant conditioning

## Abstract

As the most species‐rich vertebrate group, fish provide an array of opportunities to investigate the link between ecological interactions and the evolution of behavior and cognition, yet, as an animal model, they are relatively underutilized in studies of comparative cognition. To address this gap, we developed a fully automated platform for behavioral experiments in aquatic species, *GoFish*. *GoFish* includes closed‐loop control of task contingencies using real‐time video tracking, presentation of visual stimuli, automatic food reward dispensers, and built‐in data acquisition. The hardware is relatively inexpensive and accessible, and all software components of the platform are open‐source. *GoFish* facilitates experimental automation, allowing for customization of high‐throughput protocols and the efficient acquisition of rich behavioral data. We hope this platform proves to be a useful tool for the research community, facilitating refined, reproducible behavioral experiments on aquatic species in comparative cognition, behavioral ecology, and neuroscience.

## 1 BRIEF COMMUNICATION

We are not fish biologists, yet we recognize the importance of studying fish behavior, the most species‐rich vertebrate group (Ravi & Venkatesh, 2018), when tapping into the inner worlds of animal minds (Birch et al., 2020).

Fish provide a plethora of opportunities to investigate fundamental questions regarding how ecological pressures shape the evolution of behavior and cognition, and offer an array of less conventional model species for use in animal cognition research (Stahlman & Hoeschele, 2024).

Studying cognitive processes in animals relies on subjects learning task contingencies by experience, commonly requiring repetitive experiences or trials. To probe these cognitive processes, individuals are often then presented with alternatives to choose from, and the dynamics of the subsequent decision‐making process becomes a window into underlying hypothesized cognitive abilities. By offering subjects alternatives differing in associated features and valence, it is possible to examine how the animals attribute value to outcomes that motivate learning (e.g., Ajuwon et al., 2023; Cruz et al., 2022; Monteiro et al., 2020, 2023), how learned values are translated into action during decision‐making, and whether animals possess abstract cognitive abilities such as concept formation, including numerical (Newport et al., 2021) or temporal discriminations (Monteiro et al., 2021), for example.

For traditional model species used in comparative cognition research, including rodents, pigeons, and monkeys, there exists a range of well‐established tools to facilitate and automate behavioral testing and data acquisition, the *Skinner box* being one notable example. These operant systems enable the controlled presentation of sensory stimuli of different modalities (e.g., odors, colors, and sounds), real‐time detection of behavior (e.g., through video recording, touchscreens, operable levers, or nose ports), and the delivery of rewards to subjects (e.g., Cruz et al., 2022; Monteiro et al., 2023). The automation of behavioral testing reduces human errors and biases (Rosenthal & Fode, 1963), enhances scalable data acquisition, and improves scientific reproducibility (Baker, 2016).

However, unlike with traditional mammalian and avian models, the adoption of automated behavioral testing in cognitive research in fish and other aquatic animals, including cephalopods, is not as widespread. This could potentially limit the pace and breadth of studies investigating learning and decision‐making in a range of highly useful animal models, while also making direct interspecies comparisons difficult (but see Gatto et al. [2021] for the implementation of automated testing in guppies). Notably, while behavioral research on zebrafish has undoubtedly benefited from automated computational methods (Guilbeault et al., 2021; e.g., Manabe et al., 2013), reports of cognitively sophisticated abilities in this species are lacking, bringing into question its utility in research across multiple domains, particularly research involving cognitively demanding tasks.

Inspired by the growing trend of open‐source technology development (Lopes & Monteiro, 2021), we recently developed *GoFish*, an experimental platform for automated cognitive testing that is applicable to a range of aquatic species and is freely available for other researchers to use and expand on (Ajuwon, Cruz, et al., 2024). Here, we briefly outline *GoFish* as an accessible research tool aimed at implementing refined, automated behavioral testing in aquatic species, and suggest future avenues for its use.

**FIGURE 1:**
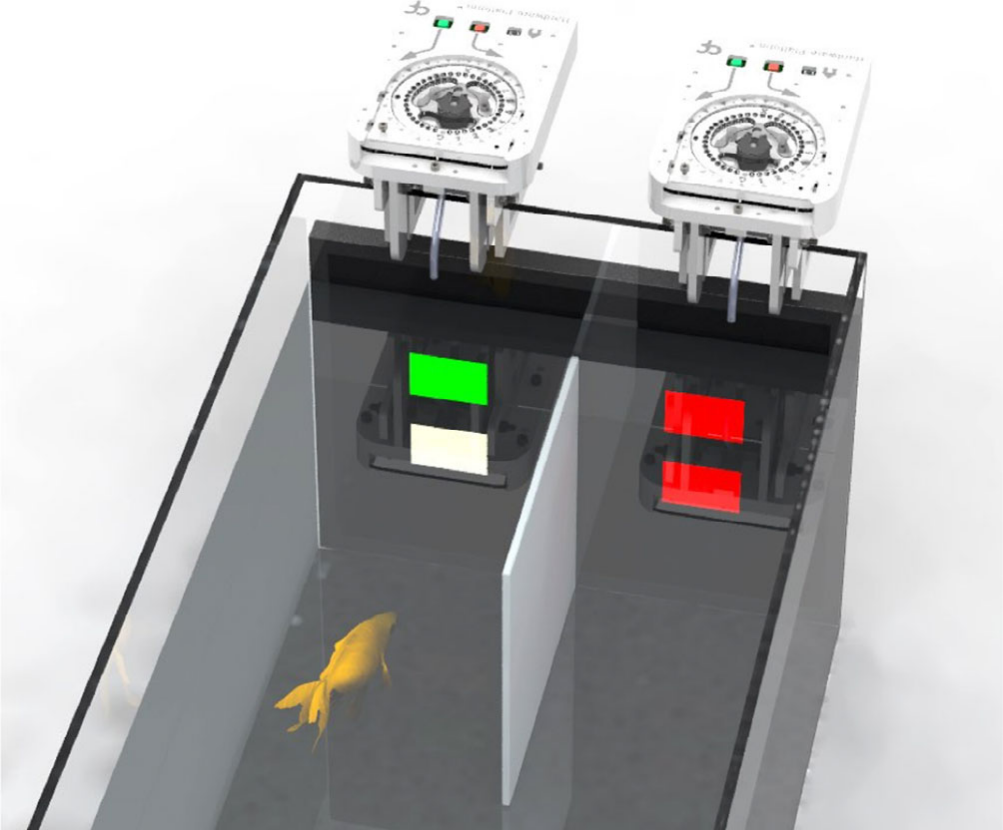
*GoFish* framework. Three‐dimensional view of a Y‐maze configuration of *GoFish*, including a computer monitor for stimulus presentation, two pellet dispensers placed either side of an opaque acrylic divider, an overhanging camera, and aquarium light (not shown). This configuration was used to test place, color, and reversal learning in goldfish (Ajuwon, Cruz et al., 2024 as well as for assessing these animals' preference in an information seeking paradigm (Ajuwon, Monteiro, et al., 2024).

*GoFish* comprises pre‐existing hardware components that are widely available and a novel, custom‐designed reward pellet dispenser whose specifications we have made available online (https://www.cf-hw.org/open-source-tools/tools/fish-feeder). As described in our original article (Ajuwon, Cruz, et al., 2024), *GoFish* consists of a monitor screen for stimulus presentation, a top‐mounted camera for behavioral monitoring and tracking, and two pellet dispensers for automatic reward delivery. The camera records subjects in a rectangular experimental aquarium with a divider installed that delineates two distinct choice zones (Figure 1). Subjects express preferences by choosing to swim into either choice zone, allowing for the dynamics of decision‐making to be recorded and cognitive processes explored. The hardware components and task implementation are controlled via *Bonsai*, a user‐friendly, open‐source software that is widely adopted for task control in behavioral neuroscience (Lopes et al., 2015, 2021; Lopes & Monteiro, 2021) and that benefits from online resources aimed at aiding researchers in task development (bonsai-rx.org).

To illustrate the utility of *GoFish* in investigating behavior and cognition, we conducted two studies of instrumental conditioning on goldfish (*Carassius auratus*, a convenient experimental model). Individuals were successfully trained to independently initiate trials by swimming to a “start zone” within the tank, then swimming into either choice zone to potentially elicit rewards. Following training, subjects were required to spatially discriminate between each choice zone, one of which was rewarded, while the other ceased to provide rewards. After successfully learning the discrimination, the rewarded side was reversed, and subjects displayed behavioral flexibility by acquiring preference for the newly rewarded side. After the spatial discrimination and reversal, subjects were then presented with a visual discrimination task, requiring them to distinguish between colored circles to elicit rewards. At the group level, goldfish were able to reliably swim into the choice zone displaying the rewarded stimulus, avoiding the unrewarded stimulus (Ajuwon, Cruz, et al., 2024).

*GoFish* facilitates efficient behavioral data acquisition, allowing users to investigate decision‐making processes at high spatial and temporal resolution, without the need for manual video annotation. Using real‐time video tracking based on color thresholding, users are provided with pre‐processed data outputs, facilitating further behavioral analysis. In the goldfish experiments described above, we examined how long it took subjects to initiate trials, and how quickly they expressed preferences, using annotated behavioral data that is automatically generated at the end of each session. In future studies, researchers could leverage the data acquisition capabilities of *GoFish* to explore a range of questions, including individual differences in learning rates and response times, speed‐accuracy trade‐offs, and sex differences in decision‐making. Furthermore, researchers could also use the animal tracking capabilities of *GoFish* to explore how subjects' movement during sessions changes through learning and in response to reward‐conditioned stimuli to gain a comprehensive picture of behavioral changes that accompany learning and problem solving.

*GoFish* can be used to efficiently answer a variety of cognitive questions. For example, since the original study, it has been used to compare the performance and preferences of goldfish (*Carassius auratus*) to published results on mammal and bird species in an information seeking paradigm (Ajuwon, Monteiro, et al., 2024).

*GoFish* is fully open‐source, and we encourage others, especially fish biologists, to re‐use, adapt, and build on top of it, tailoring it to answer their own questions. We used a two‐choice Y‐maze configuration, but other arrangements are possible, such as adapting the maze layout (e.g., radial maze), changing the position and number of feeders, or using stimuli from different sensory modalities (*Bonsai* can control a range of hardware input and output devices, promoting flexibility). In our goldfish experiments, data acquisition and behavioral task control relied primarily on video tracking using a standard camera, but in the future, researchers could obtain input data from other devices such as hydrophones, pressure sensors, or infra‐red beams that record the presence/absence of subjects and their behavior. The ability of *Bonsai* to interact with a variety of output devices means that researchers would also be able to present stimuli across a range of sensory modalities such as acoustic and vibration sensing, depending on what is most suitable for their research questions and study species. Additionally, changes can easily be made to modify the tracking software (e.g., adding background subtraction) or to implement more sophisticated routines (Dutta et al., 2023; Kane et al., 2020; Pereira et al., 2022; Walter & Couzin, 2021).

A note of caution, as raised by others (Kravitz & Laubach, 2024), is that flexible and DIY type of solutions require scientists, especially those in earlier phases of training, to invest time in developing better coding, version control, documentation practices, three‐dimensional design, and fabrication techniques, just to give a few examples. Ultimately, we believe that the benefits of enhanced experimental scalability and reproducibility will outweigh the initial time investment cost, particularly when compared to manually implemented experiments.

Studying fish cognition promises to extend our understanding of fundamental questions, including how sociality affects the evolution of behavior, how climate change impacts behavior and cognition, and the behavioral basis of colonization in invasive species. Ultimately, we believe that *GoFish* will be a valuable resource for future tool and experiment development by the fish research community, facilitating reproducible behavioral experiments on aquatic species aimed at tackling questions across comparative cognition, behavioral ecology, and neuroscience.

## AUTHOR CONTRIBUTIONS

T.M. and V.A. wrote the first draft of the paper. All authors edited and reviewed the manuscript.

## FUNDING INFORMATION

The original work this article refers to was supported by funding from the Biotechnology and Biological Sciences Research Council grant number BB/M011224/1, to V.A. The Portuguese Foundation for Science and Technology (https://www.fct.pt/en/) supported this work through a grant to T.M. (DOI: 10.54499/CEECINST/00013/2021/CP2779/CT0009) and multiannual funding to the William James Center for Research (WJCR) in the context of the project UID/04810/2020, DOI: 10.54499/UIDB/04810/2020 and 10.54499/UIDP/04810/2020.

## CONFLICT OF INTEREST STATEMENT

The authors declare no competing interests other than being some of the authors of the experimental framework highlighted in this Brief Communication.

## Data Availability

There is no data or code associated with this article. However, the code to run experiments highlighted here (and shown in Ajuwon et al., 2024) is available here: https://github.com/PTMonteiro/GoFish_Ajuwon_etal_2022.
